# Rapid and Scalable Wire-bar Strategy for Coating of TiO_2_ Thin-films: Effect of Post-Annealing Temperatures on Structures and Catalytic Dye-Degradation

**DOI:** 10.3390/molecules25071683

**Published:** 2020-04-07

**Authors:** P. Divya, S. Arulkumar, S. Parthiban, Anandarup Goswami, Tansir Ahamad, Manoj B. Gawande

**Affiliations:** 1 Nanotech Research Innovation and Incubation Centre, PSG Institute of Advanced Studies, Coimbatore-641004, India; dyaperumal@gmail.com (P.D.); sarulkumareee@gmail.com (S.A.); 2 Division of Chemistry, Department of Sciences and Humanities, Vignan’s Foundation for Science, Technology and Research (VFSTR), Vadlamudi, Guntur 522 213, Andhra Pradesh, India; ananda1911@gmail.com; 3 Department of Chemistry, College of Science, King Saud University, Riyadh-11451, Saudi Arabia; tahamed@ksu.edu.sa; 4 Regional Centre of Advanced Technologies and Materials, Palacký University, Šlechtitelů 27, 783 71 Olomouc, Czech Republic; 5 Institute of Chemical Technology Mumbai-Marathwada Campus, Jalna, Maharashtra 431203, India

**Keywords:** superhydrophilic, photocatalytic, titanium dioxide, TiO_2_, solution process technique, sol-gel ink, wire-bar technique

## Abstract

Titanium dioxide (TiO_2_) thin films were rapidly coated on Corning glass substrates from the precursor solution using the wire-bar technique at the room temperature and then post-annealed at 400, 500 and 600 °C for 1 h under atmospheric conditions. The structural, morphological, optical, wettability and photocatalytic properties of the films were studied. X-ray diffraction analysis confirmed the formation of an anatase TiO_2_ structure irrespective of the post-annealing temperatures. The optical transparency of the films in the visible range was measured to be > 70%. A water contact angle (WCA) of ~0° was observed for TiO_2_ thin-film, post-annealed at 400 °C and 500 °C. However, WCA of 40.3° was observed for post-annealed at 600 °C. The photocatalytic dye-degradation using post-annealed thin-film was investigated indicating a steady improvement in the dye-degradation percentage (from 24.3 to 29.4%) with the increase of post-annealing temperature. The demonstrated TiO_2_ thin-films deposited by wire-bar coating technique showed promises for the manufacturing of large-area cost-effective self-cleaning window glass.

## 1. Introduction

Titanium dioxide (TiO_2_) films have been widely investigated owing to their superhydrophilic, photocatalytic, anti-fogging, anti-reflection, transparent and anti-fouling properties, thereby making that class of material a prominent choice for diverse applications [1,2,3,4,5,6]. Since TiO_2_ is a nontoxic, earth-abundant, environmentally and economically friendly material [7], TiO_2_ thin-film (10–20 nm) coated surfaces have been used in self-cleaning, water and air purification, antimicrobial applications etc. [1,7,8,9]. The self-cleaning nature of TiO_2_ thin-film reduces the building maintenance cost and the expenses related to the development of the surface cleaning equipment [9]. The self-cleaning property can be achieved using both hydrophilic and hydrophobic coating materials: the former by sheeting water and the later by rolling water droplets that carries away dirt [8]. The superhydrophilic and superhydrophobic properties are attractive in self-cleaning applications and are commonly evaluated based on the water contact angles (WCAs) with the material/surface. WCAs of <5° and >150° are called superhydrophilic and superhydrophobic materials respectively [8]. The superhydrophilic material when employed on a substrate/surface leads to the formation of uniform thin layer of water upon contact and gradually carries away dust particles from the surface [9]. In contrast, hydrophobic surfaces accumulate small water droplets, leading to deterioration of transparency [7,9]. In the current context, TiO_2_ thin layers are capable of photocatalytically degrading the organic pollutants present on glass and tiles and irregular shapes on solid surfaces when irradiated with UV light or sun light [8,9]. The decomposed organic pollutant or dirt can be washed away by sheeting water without the need of additional mechanical cleaning due to its superhydrophilic nature [7,8]. Recently, the scope of superhydrophilic and photocatalytic TiO_2_ thin-films has been widened to air purification for the effective decomposition (and the subsequent removal) of nitrogen oxide (NO_x_), formaldehyde, benzene and other volatile organic compounds (VOCs) [10,11,12]. The air purification processes convert toxic gases to harmless compounds like nitrate ions that are ultimately washed into the soil and help to fertilize plants [10]. The unique wettability and non-toxic properties of TiO_2_ materials are also employed in biomedical applications where surface wettability can play an important role in protein adsorption, cell adhesion and proliferation [9]. Moreover, the antibacterial, self-cleaning, and anti-odour properties of TiO_2_ films deposited on fibres, textile, cotton, etc. also allow this material to be employed in different sectors of industry and academia [10,11,12,13].

Self-cleaning TiO_2_ thin-films have been coated using different physical and chemical vapor deposition techniques [14,15,16,17,18,19,20,21]. Recently, the solution-based approach for self-cleaning applications is attracting significant attention because of large-area scalability with cost-effective vacuum free coatings. Various techniques, including spin-coating, spray-coating, dip-coating and absorptive self-assembly can be applied to yield the final material [16,17,18,19]. For the spin-coating process, though the deposition time is shorter, it requires a large volume of precursor solution and most of this solution ends up as waste as spin-coated substrate retains only less than 5% of the loaded solution. In the case of spray-coating, dip-coating and absorptive self-assembly, a longer duration is needed for deposition of the thin-film. Though spray-coating and adsorptive self-assemble techniques are amenable to scale-up for large-area coating, they mostly require a long duration for deposition and hence are not suitable for rapid processing [17,18,19]. Thus, a technique with rapid deposition rate over a large area with minimal wastage of the precursor ink is indeed needed, and that can be satisfied by the wire-bar coating technique—a simple deposition method for large areas with rapid coating using minimal consumption of precursor ink [20,21,22]. In order to achieve superhydrophilicity, most of TiO_2_ thin-films are converted from hydrophilic to superhydrophilic using harmful UV irradiation [6,7,8,10]. Alternatively, the superhydrophilicity of the TiO_2_ thin-films can also be improved by high-temperature annealing, which removes impurities and enhances formation of a metal-oxide thin-film layer [19]. Here we focus on the deposition of TiO_2_ thin-films using a scalable process and subsequent conversion to superhydrophilic materials without UV irradiation. The TiO_2_ thin-films were coated on glass substrates at room temperature in ambient air using wire-bar coating technique and sol-gel ink. The wire coated TiO_2_ thin-films were post-annealed at 400 °C, 500 °C and 600 °C. The structural, morphological, optical, wettability and photocatalytic properties were studied for suitability in self-cleaning applications.

## 2. Results and Discussions

**Figure 1:**
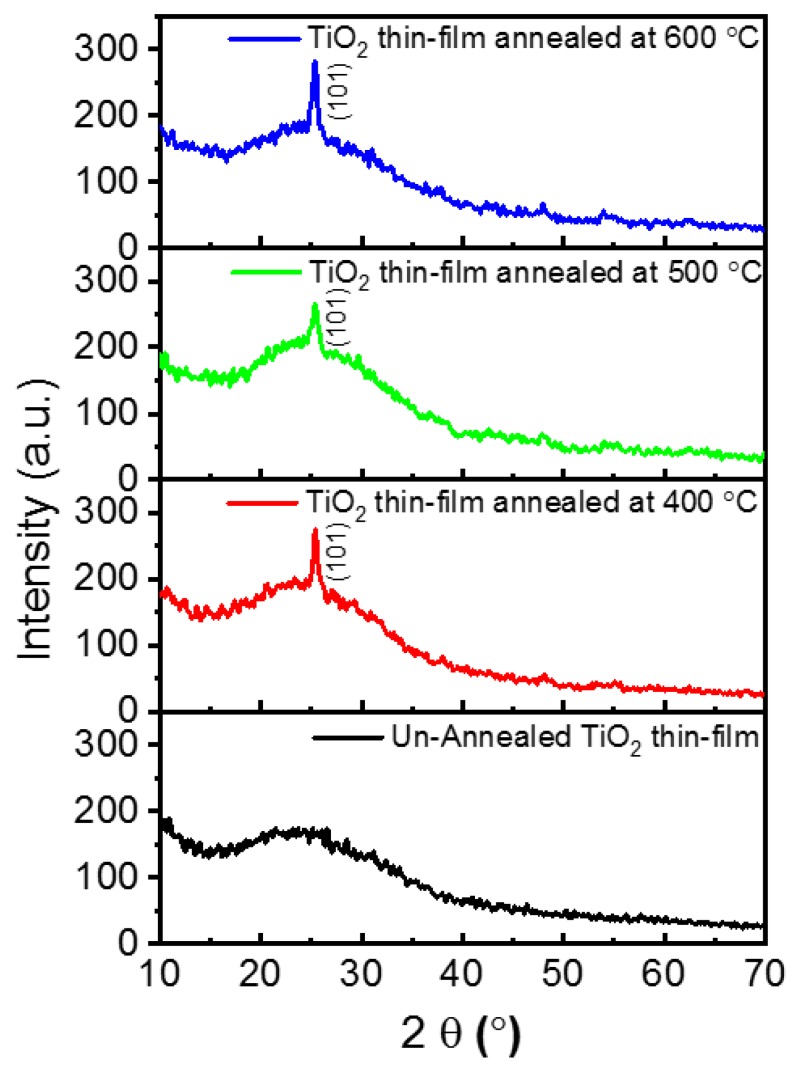
Grazing Incident X-ray Diffraction (GIXRD) patterns of TiO_2_ thin films post-annealed at different temperatures.

**Figure 2:**
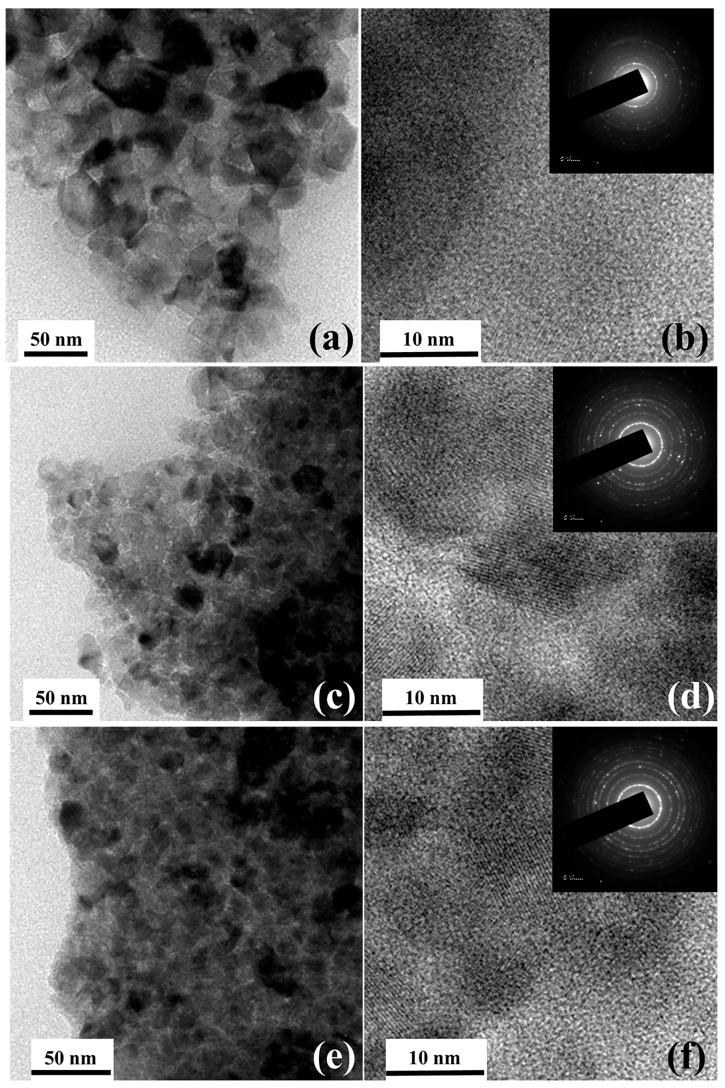
Transmission electron microscopy and high-resolution TEM (HRTEM) of TiO_2_ thin-films post-annealed at 400 °C (**a**,**b**), 500 °C (**c**,**d**) and 600 °C (**e**,**f**). The corresponding selected area electron diffraction (SAED) images of the samples are shown at insets of b, d and f.

The Grazing Incident X-ray Diffraction (GIXRD) patterns of TiO_2_ thin-films (~30 nm) post-annealed at different temperatures are shown in Figure 1. The TiO_2_ thin-films exhibit a strong diffraction peak at 2θ = 25.3° irrespective of post-annealing temperature. The obtained GIXRD peak corresponds to the (101) plane of the anatase structure (JCPDS Card no: 89-4203) [23]. The average crystal grain size is estimated from the full width at half maximum (FWHM) of the (101) peak using Scherrer formula and found to be around 20 nm without major changes [24]. Figure 2 shows the transmission electron microscopy (TEM), high-resolution TEM (HR-TEM) and selected area electron diffraction (SAED) images of TiO_2_ thin-films post-annealed at 400 °C (a, b), 500 °C (c, d) and 600 °C (e, f). The TEM images reveal agglomerated monocrystalline morphology with average crystallite size around 20 nm for the post-annealed TiO_2_ thin-films that is closely matched with the average crystallite size obtained from GIXRD pattern. The lattice spacing is measured to be 0.35 nm that conforming to the (101) crystal planes of anatase TiO_2_, and is in good agreement with the XRD pattern. From the GIXRD peak sharpening and SAED pattern, it was confirmed that the degree of crystallization increases with the increase in annealing temperature. Moreover, the corresponding SAED pattern reveals the polycrystalline nature.

**Figure 3:**
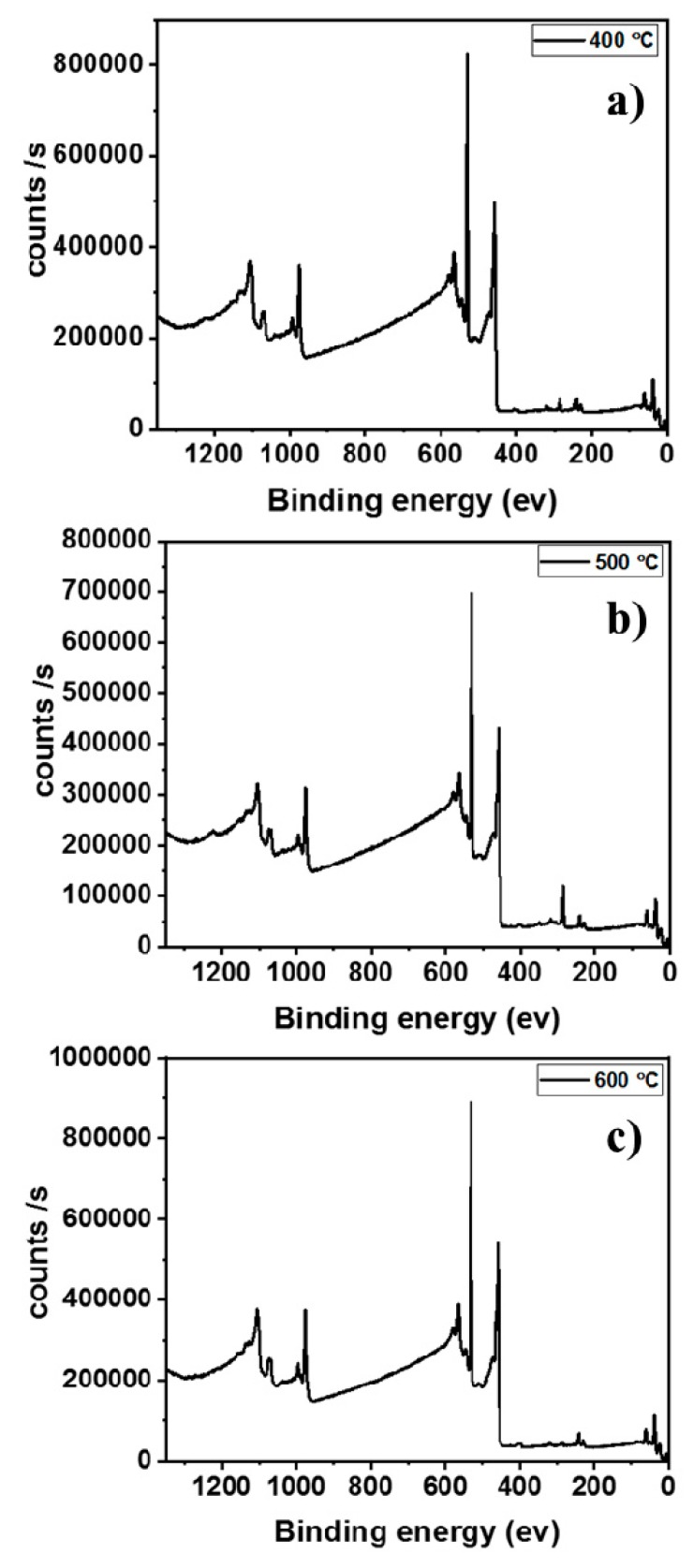
X-ray photoelectron spectroscopy (XPS) survey spectra of TiO_2_ thin-films post-annealed at different temperatures. (**a**) TiO_2_ thin-films post-annealed at 400 °C; (**b**) TiO_2_ thin-films post-annealed at 500 °C; (**c**) TiO_2_ thin-films post-annealed at 600 °C.

**Figure 4:**
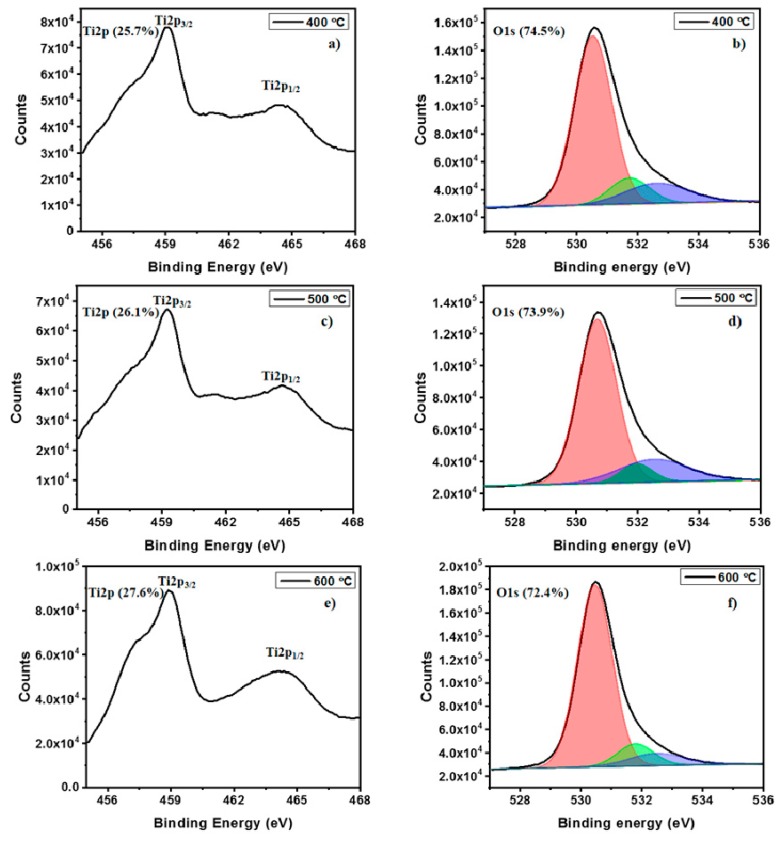
Deconvoluted XPS spectra of TiO_2_ thin-films post-annealed at different temperature. (**a**) The Ti 2p spectra, 400 °C; (**b**) The O 1s spectra, 400 °C; (**c**) The Ti 2p spectra, 500 °C; (**d**) The O 1s spectra, 500 °C; (**e**) The Ti 2p spectra, 600 °C; (**f**) The O 1s spectra, 600 °C.

The elemental composition of TiO_2_ thin-films post-annealed at 400, 500 and 600 °C were obtained using X-ray photoelectron spectroscopy (XPS). XPS analysis confirmed the presence of Ti and O in each sample and the survey spectrum of the samples were shown in Figure 3. The obtained deconvoluted spectra of Ti and O along with their elemental concentrations are shown in the Figure 4. The Ti 2p spectra consists of two peaks at 459.2 eV and 464.5 eV assigned to the Ti 2p_3/2_ and Ti 2p_1/2_. The obtained peaks for Ti 2p spectra are closely matching with previously reported TiO_2_ [23]. The O 1s peaks were deconvoluted into three individual sub-peaks. The dominant peak at 529.8 eV (orange) arises from O atoms bonded to metal ions (M-O-M) in the lattice. The peak at 530.4 eV (green) is attributed to oxygen vacancy and the peak at 532.2 eV (blue) can be assigned to metal hydroxide (M-OH). From the area occupied by the deconvoluted oxygen peaks, it was noted that annealing at higher temperature greatly influences the M-O-M bonding, increasing the crystallinity at higher temperature. Also, the M-OH bonding in the samples gradually decreases as annealing temperature increases due to conversion of M-OH to M-O-M possibly via condensation.

**Figure 5:**
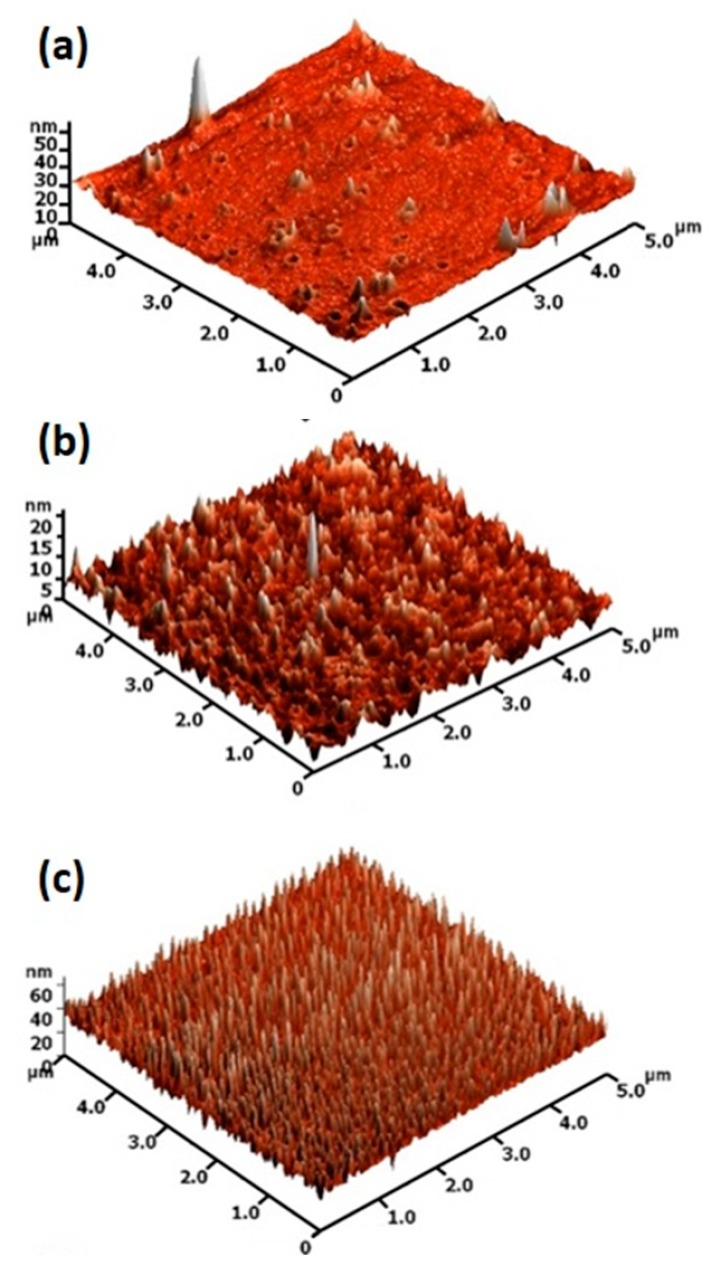
AFM surface morphology images of the TiO_2_ thin film post-annealed at (**a**) 400 °C, (**b**) 500 °C and (**c**) 600 °C.

The atomic force microscopy (AFM) surface morphology images of the post-annealed TiO_2_ thin-films are shown in Figure 5. The AFM images indicate a smooth surface with a few defects along with scattered grains for post-annealed TiO_2_ thin-film at 400 °C. In contrast, nearly defect-free agglomerated grains were observed for TiO_2_ thin-film post-annealed at 500 and 600 °C. The surface roughness of post-annealed TiO_2_ thin-film increased with increasing annealing temperature. This observation along with the increase in M-O-M bonding as shown in XPS analysis showed that increasing annealing temperature resulted in enhanced crystallinity of the films. The root- mean-square (RMS) roughness values of 0.92, 8.74 and 34.82 nm were observed for TiO_2_ thin-film post-annealed at 400 °C, 500 °C and 600 °C.

**Figure 6:**
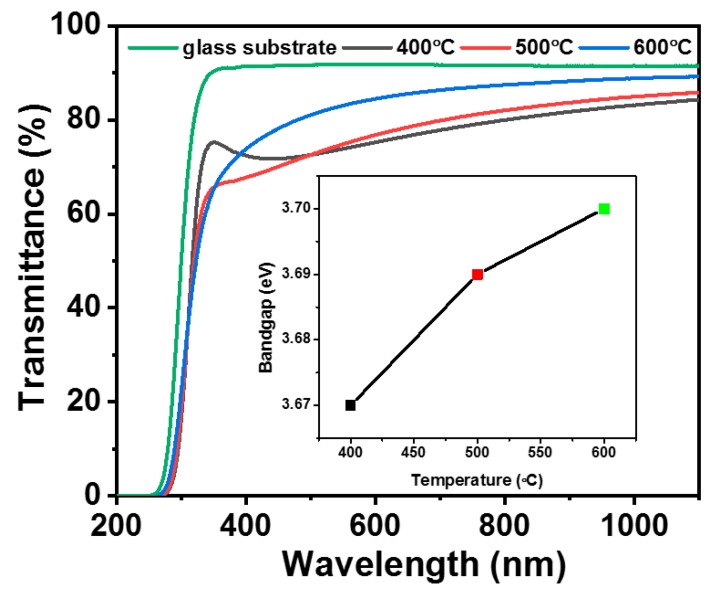
Transmittance spectra of post-annealed TiO_2_ thin-films. (inset: Shift in band gap with annealing temperature).

The transmittance spectra of post-annealed TiO_2_ thin-films in the wavelength range of 200–1100 nm are shown in Figure 6. The unannealed TiO_2_ thin-film has a transmittance of ~81%, whereas all the TiO_2_ thin-films after annealing shows > 70% optical transmission in the visible region, without strong interference fringes, which is sufficient for self-cleaning window applications. The bandgap of these films was calculated using transmittance spectra by Tauc relation [25]. The bandgap values were obtained by extrapolating the linear portion of the plot *(αhυ)^1/2^* versus *hυ* to *(αhυ)^1/2^* = 0. The calculated band gap of TiO_2_ thin-film post-annealed at 400 °C was around 3.67 eV as shown in Figure 6 (inset) with a slight increase in bandgap with an increase in annealing temperature. The slight shift could be attributed to the increase in crystallinity with annealing temperature.

**Figure 7:**
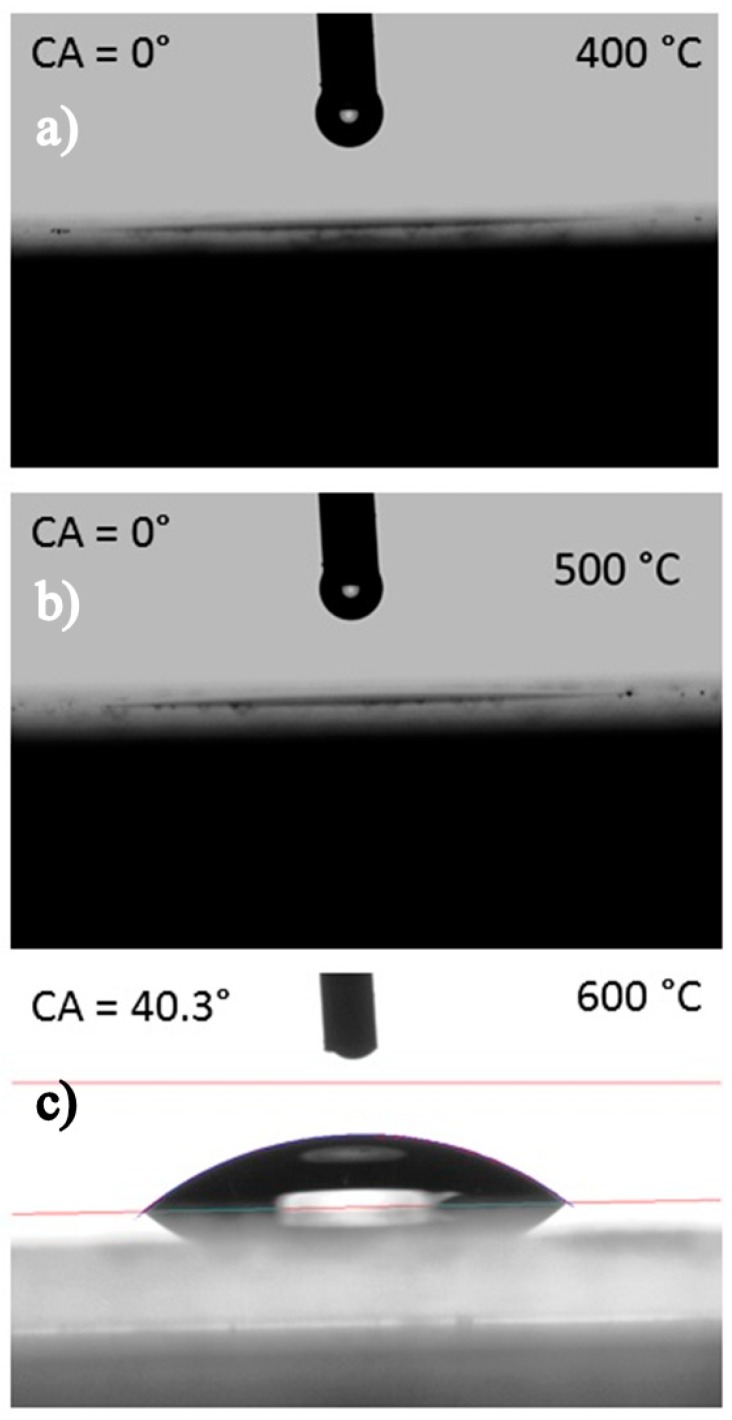
Water contact angle (WCA) of TiO_2_ thin-films post-annealed at (**a**) 400 °C, (**b**) 500 °C and (**c**) 600 °C.

The superhydrophilicity of the surfaces was estimated through the static water contact angle between a water droplet and TiO_2_-coated glass surfaces. Figure 7 shows the water contact angle (WCA) of post-annealed TiO_2_ thin-films. The films annealed at 400 °C and 500 °C show the lowest WCA of θ~0^°^. The water droplet spreads completely and instantaneously on the film of samples post-annealed at 400 and 500 °C. The post-annealing process removes the organic contaminates present on the surface of TiO_2_ which effectively transforms the wettability of the TiO_2_ surface from hydrophilic to superhydrophilic. However, a WCA of 40.3° was observed in post-annealed TiO_2_ thin-film at 600 °C, presumably owing to low M-OH bonds on the surface and high roughness of the surface as observed in the atomic force micrograph. As seen from the surface topography analysis, the closely packed pillarlike structures on the TiO_2_ thin-film post-annealed at 600 °C could trap air between them when water was added on top preventing superhydrophilicity.

**Figure 8:**
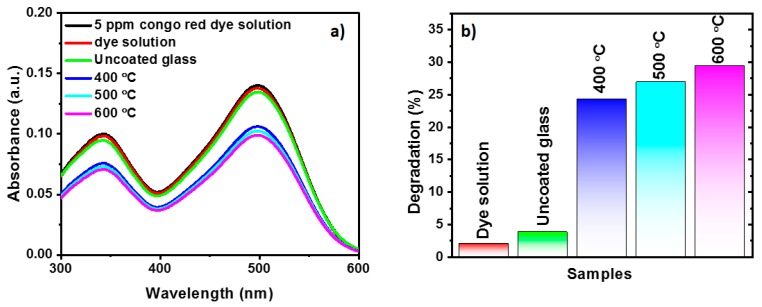
Photocatalytic activity of TiO_2_ thin-film coated glass post-annealed at different temperature (**a**) Congo-red absorbance, (**b**) percentage of degradation.

The UV-Vis spectrophotometer was used in the absorption mode to calculate the degradation percentage (%) of Congo red dye solution before and after UV light irradiation. The Congo red (CR) dye solution exhibits two absorption peaks at 340 nm and 498 nm as shown in Figure 8a. The change in highest absorption peak intensity at 498 nm was used to calculate the dye degradation percentage using the following expression [26,27].

X % = (C_0_ − C) /C_0_ × 100 (1)

where C_0_ is the initial absorbance of dye solution and C is the absorbance of dye solution after UV irradiation.

The photocatalytic degradation of Congo red dye solution was evaluated for TiO_2_ thin-film-coated glass substrates as a function of post-annealing temperature and the same is shown in Figure 8. It was noted that, after the immersion of samples for 2 h in dye solution, no major change was observed in the absorbance of dye solution. Hence, the drop in the absorbance of dye solution after UV irradiation was attributed to degradation of dye molecules in solution. The photo degradation of the dye solution and glass substrates was negligible when compared to TiO_2_- coated glass substrates. In contrast, the intensity of the Congo-red (CR)absorption peak significantly decreased for TiO_2_ thin-film with increase in post-annealing temperature. Dye degradation percentages of 24.3, 27.0 and 29.4 were exhibited for TiO_2_ thin-films post-annealed at 400 °C, 500 °C and 600 °C. The highest dye degradation percentage of 29.4 was observed for TiO_2_ thin-film post-annealed at 600 °C. The enhanced photocatalytic performance may arise from the crystalline nature of TiO_2_ thin-film as well as the increase in effective contact area. The comparison of the obtained results with some of the previously reported results is shown in Table 1.

## 3. Experimental

### 3.1. TiO_2_**** Ink Synthesis

The TiO_2_ ink was synthesized using a sol-gel method as reported in [31,32,33]. The TiO_2_ sol-gel ink was synthesized using a three-necked, 250 mL, round-bottom flask. One of the necks of the three-necked flask was connected with a condenser and other two were sealed with rubber septa. The two sealed necks were used for injecting solutions and nitrogen-gas (N_2_) purging respectively. The nitrogen gas was purged into the round bottom flask until the completion of the experiment. The flask was heated to 120 °C for 2 h in the silicone oil bath to remove inside moisture from the flask. Once the three-necked flask reached room temperature (~30 °C) titanium (IV) isopropoxide (Ti[OCH(CH_3_)_2_]_4_), aldrich, >97.0%, 3 mL of 0.5 M), 2-methoxyethanol (CH_3_OCH_2_CH_2_OH, Sigma-Aldrich, St. Louis, MO, USA, 99.9%, 20 mL) and monoethanolamine (NH_2_CH_2_CH_2_OH, Aldrich, 99%, 1.2 mL of 1 M) solutions were injected respectively. The solution was stirred at room temperature for 1 h. The mixed solution was heated at 80 °C and maintained for 1 h, then subsequently heated to 120 °C and maintained for 1 h with constant stirring. After the completion of the reaction, N_2_ purging was maintained until cooling it to 30 °C. Finally, methanol (CH_3_OH) solvent was added to prepare 20 mL of TiO_2_ ink (0.5 M). The prepared TiO_2_ ink was further diluted using methanol to 0.1 M concentration for the wire-bar coating process. The prepared ink was stable for several months in tightly closed glass container.

### 3.2. Wire-Bar Coating Process

**Figure 9:**
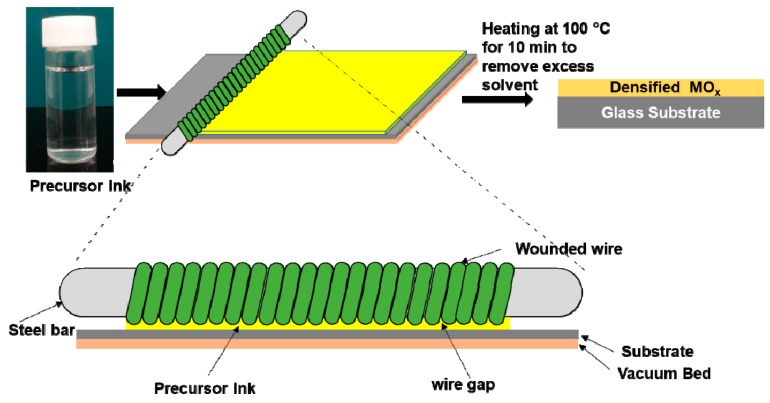
Schematic of wire-bar coating process.

The detail of wire-bar coating technique is given in Figure 9. In short, Corning glass substrates (25 mm × 75 mm) (Sigma-Aldrich, St. Louis, MO, USA) were cleaned with DI water, acetone, isopropanol and again DI water respectively for 20 min (mins) in an ultrasonic bath and dried using a nitrogen gas purging. The cleaned glass substrates were UV/ozone-treated for 20 min using UV ozone cleaner (PSDP-UV8T, Nova Scan, IA, USA) to remove organic residues and enhance wettability. The cleaned glass substrate was placed on vacuum bed that rigidly gripped on the aluminum metal plate. A 1.3 cm-diameter, 30 cm-long rod with a ~100 µm-diameter-wire-wound bar was used for the wire-bar coating process. The wire-wound bar was placed on the top of glass substrate and locked using a metal fastener. A volume of 15 µL of diluted TiO_2_ ink was dropped across the glass substrate. The movement of the bar was maintained at 15.3 cm/sec. Then the TiO_2_-ink-coated glass substrate was kept at room temperature for 30 min and post-annealed at 400, 500 and 600 °C in atmospheric conditions for 1 h.

### 3.3. Characterization

The thickness of TiO_2_ thin-films on glass substrates was determined with an Alpha-Step D-600 Stylus Profiler. The grazing incidence X-ray diffraction (GIXRD) patterns of TiO_2_ coated glass substrates were examined using PANalytical Empereon (Almelo, Netherlands) with Cu K_α_ radiation (λ = 1.5418 Å) and incidence angle of 1.5°. Binding energies and elemental compositions of the films were analyzed using X-ray photoelectron spectroscopy (Thermo scientific Waltham, MA, USA) with Al K_α_ source. For each sample, before the experiment, a 10 nm thickness was etched using Ar^+^ ion sputtering to remove the carbon contamination on the surface layer due to air exposure. Atomic force microscopy images were captured using an NT-MDT (tapping mode, NTEGRA Prima, Russia) with silicon nitride tip. High-resolution transmission electron microscopy (HRTEM) and fast Fourier transform (FFT) images were obtained using a JEOL JEM 2100 HRTEM. For TEM sample preparation, the coated TiO_2_ thin-film was removed from the glass substrate by gentle scratching and immersed it in ethanol solution. Finally, a few drops of that ethanol solution were added on carbon coated copper grid then dried at 150 °C in atmospheric conditions for 30 min. The UV–visible–near-infrared transparency studies were carried out using a double beam UV-Vis-NIR spectrophotometer (UV-1800 SHIMADZU, Japan) from 200–1100 nm at 1 nm intervals. The wettability of the coatings was evaluated using contact angle measurement system (OCA-15EC, Data Physics, Germany at atmospheric conditions. A 3 μL DI water was dispensed to the TiO_2_-coated glass substrate from an automatic dispenser with a syringe. The contact angles were measured by fitting the image of the water droplet in a Laplace-Young model [1].

### 3.4. Photocatalytic Activity Testing

5 mg of Congo red dye was dissolved in 1 liter of deionized (DI) water. 30 mL of this dye solution was taken in a 50 mL beaker for dye degradation studies. Uncoated glass and TiO_2_-thin-film-coated glass substrates (2 × 2.5 cm^2^) were immersed in the 30 mL of Congo red dye solution and stored in the dark for 2 h. The UV (λ = 365 nm, 125 W) light setup from HEBER, Scientific Instrument (India) was used for irradiation. The beakers containing dye solution with substrates and without substrate were placed 10 cm away from UV light source. After 5 h of UV irradiation, the dye solutions were analyzed to study the photocatalytic degradation [27]. The dye degradation percentage (%) was calculated from the absorption spectra using the formula (C_o_ − C) × 100/C_o_ where C_o_ is the initial absorption value of the dye and C is the absorption value of the dye after irradiation.

## 4. Conclusions

In the present work, TiO_2_ thin films were deposited on Corning glass substrates by the wire-bar coating technique at room temperature followed by post-annealing at 400 °C, 500 °C and 600 °C for 1 h. The XRD patterns of post-annealed films showed an anatase crystalline structure irrespective of post-annealing temperature. The AFM images indicated that post-annealing significantly affects surface roughness. The post-annealed films were highly transparent in the visible region (>70%). The optical bandgap increased slightly with increasing annealing temperature. The coating exhibits superhydrophilic nature when post-annealed at 400 °C and 500 °C. The photocatalytic activity test showed dye degradation percentages of 24.3, 27.0 and 29.4 for TiO_2_ thin-film post-annealed temperature at 400 °C, 500 °C and 600 °C. The combination of the best superhydrophilic, transparent and photocatalytic properties were observed for TiO_2_ thin-film post-annealed at 500 °C and this approach seemed promising for developing various nano-coating for diverse applications.

## Figures and Tables

**Table 1:** Comparison of dye degradation activity of TiO_2_ thin-films.

Material	Dye used and Duration	Degradation %	Reference
TiO_2_ thin-film (wire-bar coating)	Congo red	29.4% at 5 h for TiO_2_ thin-film annealed at 600 °C.	This work
Ceria/Au membrane (electrospinning)	Methylene blue	90% in 24 h	[28]
Au/ZnO mats (electrospinning)	Methylene blue	35% at 5 h	[29]
TiO_2_ Nanopowder	Methylene blue	~30% at 5 h for TiO_2_	[30]
